# Plasma exchange with albumin replacement and disease progression in amyotrophic lateral sclerosis: a pilot study

**DOI:** 10.1007/s10072-021-05723-z

**Published:** 2021-11-18

**Authors:** Mónica Povedano, Andrés Paipa, Miquel Barceló, Michael K. Woodward, Sandra Ortega, Raúl Domínguez, Maria Esperança Aragonés, Raquel Horrillo, Montserrat Costa, Antonio Páez

**Affiliations:** 1 Department of Neurology, Hospital Universitari de Bellvitge, L’Hospitalet de Llobregat, Barcelona, Spain; 2 Grifols Bioscience Research Group, Grifols, Barcelona, Spain; 3 Department of Apheresis, Banc de Sang i Teixits, Barcelona, Spain

**Keywords:** Amyotrophic lateral sclerosis, Plasma exchange, Albumin, Cognitive function, Motor dysfunction

## Abstract

**Background:**

Plasma exchange (PE) is used to treat a range of neurological disorders. Based on results demonstrated in Alzheimer’s disease, we theorized that PE with albumin replacement (PE-A) might alter the metabolic profile of plasma and cerebrospinal fluid in patients with amyotrophic lateral sclerosis (ALS) by removing disease-inducing molecules. The aim of this study was to evaluate the effect of PE-A on disease progression in ALS.

**Methods:**

In this open-label, non-controlled, single-arm, prospective pilot study, 13 adults with ALS had 6 months’ treatment with PE-A 5% and 6 months’ follow-up. Primary endpoints were changes from baseline in the Amyotrophic Lateral Sclerosis Functional Rating Scale-Revised (ALSFRS-R) score and forced vital capacity (FVC) through 48 weeks. A post hoc analysis compared individual patient data with the expected ALSFRS-R progression slope.

**Results:**

The median ALSFRS-R score declined throughout the study, although the rate of decline was slower than expected in seven patients at treatment end and in five patients at study end. Six patients remained in the same baseline slope progression category, and four patients improved their slope category at treatment end. Median FVC decreased significantly during the study. Treatment was well tolerated. Of 330 PE-A procedures, 0.9% were associated with potentially related adverse events.

**Conclusion:**

Although functional impairment progressed, about two-thirds of patients showed a slower than expected rate of decline at treatment end. Most patients had unaltered (54.5%) or reduced (36.4%) ALSFRS-R slope progression at treatment end. Further evaluation of PE-A in controlled studies involving more patients is warranted.

**EudraCT number:**

2013-004842-40.

**Trial registration:**

ClinicalTrials.gov identifier: NCT02479802.

## Introduction

Amyotrophic lateral sclerosis (ALS) is a neurogenerative disease characterized by progressive loss of the upper and lower motor neurons [1]. Patients develop progressive weakness that spreads within and between body regions [2, 3]. Cognitive and behavioural changes occur in up to 50% of patients [1]. Although a modest proportion (~ 20%) of patients with ALS survive beyond 5 years, more than half die within 30 months of symptom onset [4], usually due to respiratory failure [1].

The pathophysiology of ALS is not fully understood but is thought to reflect a complex interaction of genetic mutations and environmental factors that leads to dysfunction of various molecular pathways affecting motor neurons, resulting in neurodegeneration [1, 5]. Among these pathways, oxidative stress-induced lipid peroxidation may alter lipid metabolism and signalling leading to the accumulation of toxic compounds and disruption of the blood–brain barrier [6–14]. Neuroinflammation, which is characterized by macrophage and T lymphocyte infiltration and appearance of reactive astrocytes and microglia, is also strongly implicated in the pathogenesis of ALS, providing a potential therapeutic target aimed at modulating the inflammatory environment to preserve remaining motor neurons [15, 16].

Treatment options for patients with ALS are limited. The only disease-modifying drug approved for use in Europe is riluzole, which shows modest efficacy [17]. Edaravone is approved in Japan and USA but not in Europe [1]. Current treatment is aimed mainly at symptom relief, highlighting the need for drugs that can alter the disease course.

A few small studies/case series conducted about 40 years ago evaluated plasma exchange (PE) to treat patients with ALS [18–20]. Therapeutic PE is an extracorporeal blood purification process used to remove pathogenic antibodies and other large molecular-weight substances from the blood. Although PE was shown to be generally safe in patients with ALS, there was little evidence of efficacy, which was possibly due to methodological limitations. Since then, standardized recruitment criteria and validated efficacy endpoints for ALS have become available for use in clinical trials [21]. Moreover, the safety of plasmapheresis-based procedures has improved greatly. PE is currently used to treat a range of neurological disorders with an inflammatory component [22–25].

A 5% human serum albumin solution is commonly used as a replacement solution during PE. In addition to maintaining the colloid osmotic pressure of plasma, albumin has several other functions. It is involved in the transportation of molecules (e.g. hormones, fatty acids, metabolites and drugs) in the blood; has a role in the regulation of microvascular permeability and has antioxidant, antithrombotic and anti-inflammatory effects [26–28]. Animal models have suggested that albumin provides neuroprotection through multiple actions which include reducing cerebral oedema, increasing tissue perfusion, preventing thrombosis and exerting antioxidant activity [26, 28–30]. As investigated in Alzheimer’s disease [31–33], PE with albumin replacement (PE-A) may alter the metabolic profile of the plasma and cerebrospinal fluid in ALS patients by removing disease-inducing molecules and providing benefits related to the multiple functions of albumin.

On these premises, we considered it appropriate to re-examine the potential therapeutic role of PE-A in patients with ALS. This pilot study was undertaken to evaluate the effect of PE-A on disease progression in patients with ALS, as reflected by measures of functional impairment and respiratory function. Other assessments included the effects of PE-A on cognitive function, the characteristics of albumin and of a lipid peroxidation marker and the safety and tolerability of albumin and the PE procedure.

## Methods

This prospective, open-label, non-controlled, single-arm pilot study was conducted in a single centre at the Hospital Universitari Bellvitge in Barcelona, Spain. Study duration was 12 months, which consisted of 6 months’ PE-A and 6 months’ follow-up.

Eligible for inclusion were men and women aged 18 to < 70 years with a definite, possible or probable diagnosis of ALS according to El Escorial/Airlie House criteria [2], who had experienced initial ALS symptoms within 18 months of recruitment/consent and had a forced vital capacity (FVC) > 70% of predicted value. Exclusion criteria were concurrent neurodegenerative diseases or other non-ALS diseases associated with motor neuron dysfunction; pre-existing clinically significant lung disease not due to ALS; unsuitability for repeated PE procedures (e.g. problematic peripheral vein access, history of adverse reactions to albumin solution or blood products, presence of heart disease such as congestive cardiac failure); renal dysfunction (creatinine concentration > 2 mg/dL); presence of behavioural disorders requiring pharmacological intervention with < 3 months’ stable treatment; and any condition that may have complicated compliance with the study protocol.

The treatment period consisted of an intensive phase involving two PE-A sessions per week for 3 weeks (6 procedures), followed by a maintenance phase involving one PE-A session per week for 21 weeks (21 procedures), for a total of 27 procedures.

PE was performed using a 5% albumin solution (Albutein® 5% intravenous infusion; Grifols). One plasma volume was processed per procedure. Replacement volume was calculated according to an individual patient’s gender, weight and haematocrit. Peripheral access was used, and all procedures were performed at a preferred rate of 40–100 mL/min using a continuous-flow blood cell separator. The total time per procedure was between 1.5 and 3 h, including preparation time and patient monitoring after completion.

Patients continued treatment with pre-existing established medications, including riluzole, in adherence with recommendations that riluzole not be administered immediately before or after PE-A and that antiplatelet medications not be administered on the day of a procedure.

Scheduled visits were at baseline, at weeks 4, 12 and 24 during treatment and at weeks 25, 36 and 48 during follow-up. The Amyotrophic Lateral Sclerosis Functional Rating Scale-Revised questionnaire (ALSFRS-R) (range, 0 to 48, with higher scores indicating better function) [34], lung function tests (FVC and FVC [%]) and electromyography (EMG) were performed at baseline and at weeks 4, 12, 25, 36 and 48. Surface EMG was used to assess motor-evoked potentials in the distal muscles of the upper limbs (thenar eminence stimulating median nerve and hypothenar eminence stimulating cubital nerve, both at the wrist) and in the ankle dorsiflexor muscles in the lower limbs (anterior tibialis stimulating common peroneal nerve at the fibular head). The ALS-Cognitive Behavioral Screen (ALS-CBS) [35] and ALS Assessment Questionnaire 40 (ALSAQ-40) [36] were administered at baseline and at weeks 25 and 48.

Albumin functional capacity in plasma was measured at baseline and at weeks 4, 12, 24, 36 and 48. Total albumin concentration was determined by immunonephelometry. The binding capacity of albumin to fatty acids was determined by electronic paramagnetic resonance, incubating samples with increasing concentrations of the spin probe 16-doxylstearic acid and expressing the results by the dissociation constant K_d_. The lipid peroxidation marker 8-iso-prostaglandinF2α (8–iso-PGF2α) was measured in plasma by mass spectrometry at baseline and at weeks 12 and 24.

Primary efficacy variables were changes from baseline in the ALSFRS-R overall score and FVC. Secondary efficacy variables were changes from baseline in ALS-CBS and ALSAQ-40 scores, biomarkers and EMG profile.

A post hoc analysis compared individual patient outcomes with the expected ALSFRS-R progression slope and classified patients as slow, normal or fast progressors. Based on usual survival of between 3 and 5 years from symptom onset in ALS and a usual decrease of one point per month in the ALSFRS-R overall score [21], slow progressors were defined as patients with an ALSFRS-R slope less than − 0.8 points/month, normal progressors as those with an ALSFRS-R slope between − 0.8 and − 1.33 per month and fast progressors as those with an ALSFRS-R slope greater than − 1.33 points/month.

Safety variables, including adverse events (AE), vital signs and clinical laboratory parameters (coagulation, blood count, biochemistry, serology), were assessed at each visit.

### Statistical methods

Continuous variables are reported as mean and standard deviation (SD) or as median and interquartile range (IQR). Categorical variables are presented as absolute (*n*) and relative (%) frequency. Changes from baseline in primary efficacy variables, and changes from baseline in ALSFRS-R scores for functional subdomains, are summarized by visit. Differences between baseline and subsequent visits were analysed using the student *t* test (normal distribution) or a nonparametric Wilcoxon signed-rank test.

Secondary efficacy variables and safety variables are summarized by visit. Albumin and 8-isoPGF2α data are expressed as median and IQR. Paired comparisons between data before and after PE-A were analysed using the Wilcoxon test.

The primary efficacy analysis was performed in the evaluable population which included patients who had received at least one PE procedure and had at least one baseline measurement and subsequent measurement of at least one primary efficacy variable.

As this was a pilot study, no formal calculation of sample size was performed. A sample size of 10 was considered adequate to meet the study objectives and to verify proof of concept for use of PE-A in patients with ALS.

### Standard protocol approvals and patient consent

The study was conducted in accordance with the principles of the Declaration of Helsinki and was approved by an independent institutional review board. All participants provided written informed consent to participate.

## Results

### Patient disposition

**Fig. 1:**
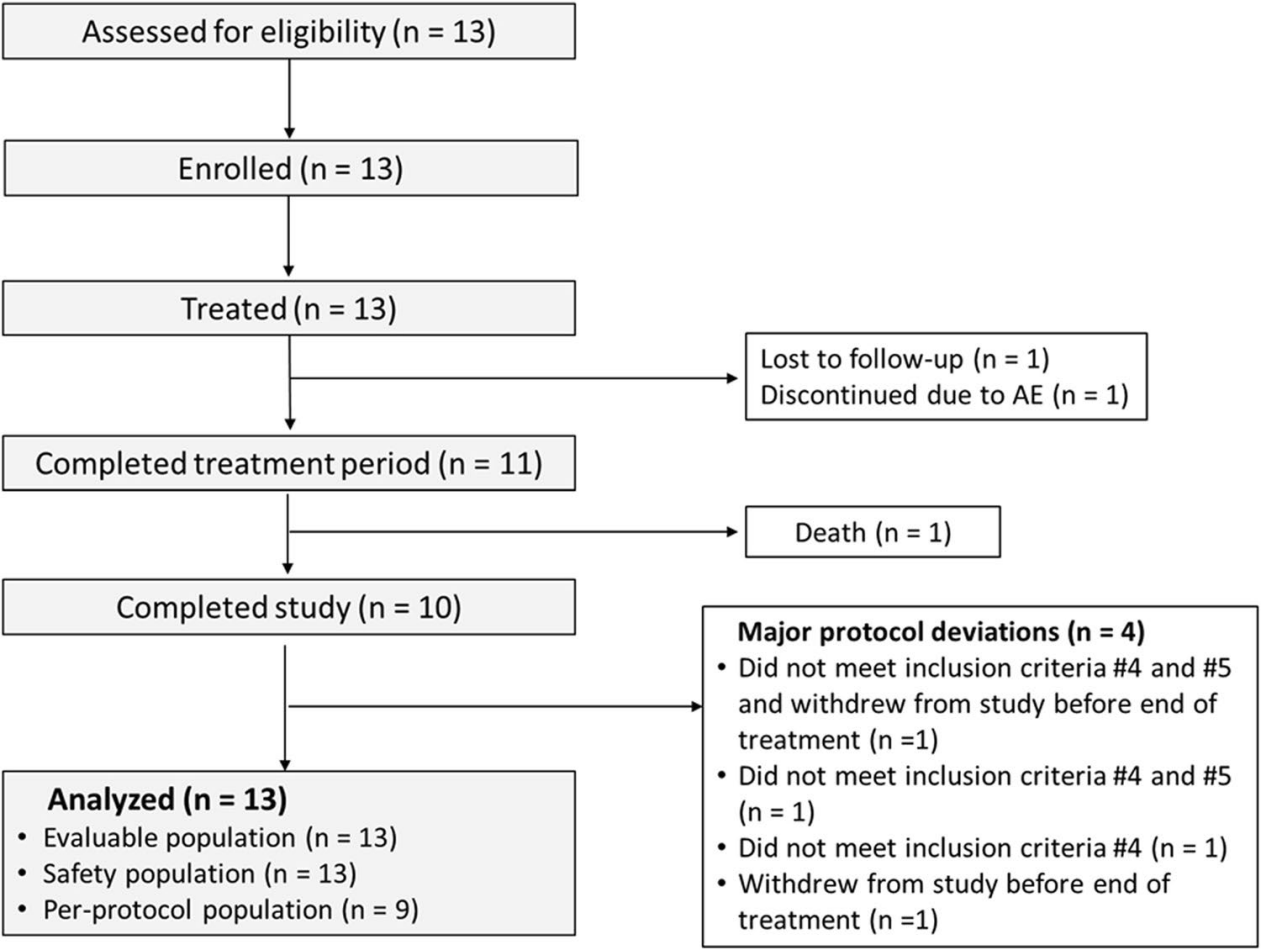
Patient disposition

Thirteen patients were enrolled into the study between November 2014 and June 2016. Three more subjects were enrolled than originally planned because of protocol violations. Three patients exceeded the 18-month time limit since first symptom of ALS of whom two had a baseline FVC < 70% of predicted. All 13 patients were included in the evaluable and safety populations (Fig. 1). Eleven patients completed planned treatment at week 24. Ten patients completed the study at week 48. The three patients who violated inclusion criteria (one of whom discontinued due to an AE during the treatment phase) and one patient who withdrew before completing half the scheduled treatment were excluded from the per-protocol population.

### Baseline characteristics

Characteristics of study participants at baseline are summarized in Table 1. There were nine men (69.2%) and four women (30.8%). Mean ± SD age was 48.9 ± 9.9 years. Median (IQR) time since ALS symptom onset and ALS diagnosis was 13.6 (8.1, 17.8) and 1.9 (1.1, 14.8) months, respectively. All patients had definite (*n* = 6, 46.2%) or probable (*n* = 7, 53.8%) ALS according to El Escorial/Airlie House criteria.

**Table 1:** Baseline characteristics of the study participants (*n* = 13)

Characteristic	Value			
Age (years), mean (SD)	48.9 (9.9)			
Gender M/F, *n* (%)	9 (69.2)/4 (30.8)			
Weight (kg), mean (SD)	67.9 (13.3)			
Height (cm), mean (SD)	168.6 (8.5)			
Time since symptom onset (months), median (IQR)	13.6 (8.1, 17.8)			
Time since diagnosis (months), median (IQR)	1.9 (1.1, 14.8)			
Region affected	First body part	At baseline		
		UMN (clinical)	LMN (clinical)	LMN (EMG)
Bulbar, *n* (%)	5 (38.5)	7 (53.8)	7 (53.8)	12 (92.3)
Left upper extremity, *n* (%)	1 (7.7)	11 (84.6)	11 (84.6)	13 (100)
Right upper extremity, *n* (%)	4 (30.8)	12 (92.3)	11 (84.6)	13 (100)
Trunk, *n* (%)	0 (0)	5 (38.5)*	5 (38.5)	13 (100)
Left lower extremity, *n* (%)	2 (15.4)	9 (69.2)	7 (53.8)	13 (100)
Right lower extremity, *n* (%)	1 (7.7)	8 (61.5)	7 (53.8)	13 (100)

^*^Four patients (30.8%) were not assessed for UMN affecting the trunk

*EMG*, electromyography; *IQR*, interquartile range; *LMN*, lower motor neuron; *SD*, standard deviation; *UMN*, upper motor neuron

Body regions affected by upper motor neuron dysfunction (assessed clinically) and lower motor neuron dysfunction (assessed clinically and by EMG) at baseline are shown in Table 1. There were the 5 bulbar onset (38.5%) and 8 spinal onset (61.5%) participants. All patients showed both upper and lower motor neuron signs according to El Escorial criteria.

Median total albumin concentration in the cohort at baseline was 42.70 (range, 38.99 − 53.45) mg/mL which was within the accepted normal range.

### Functional impairment: ALSFRS-R

**Fig. 2:**
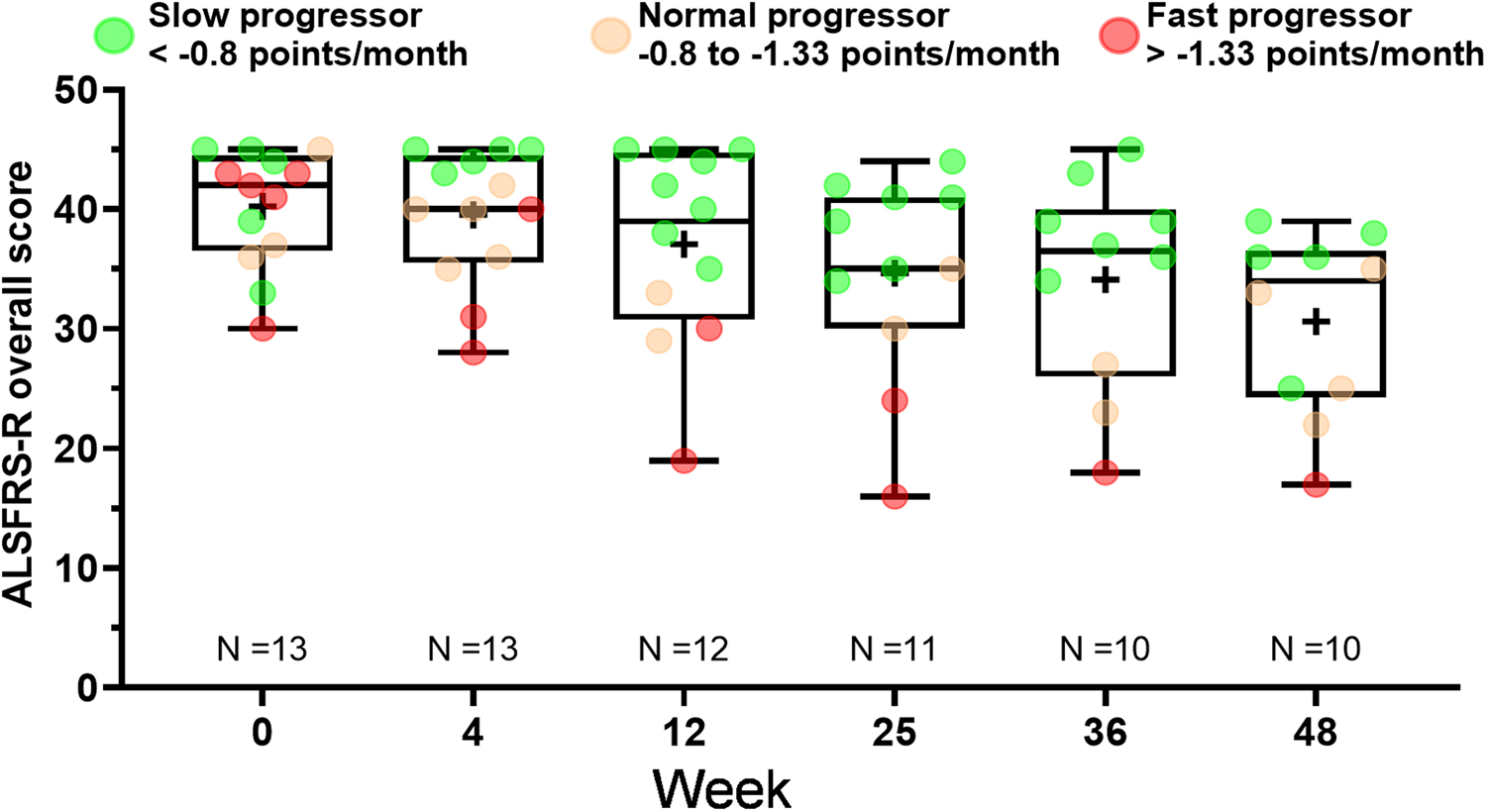
Progression of disability: Amyotrophic Lateral Sclerosis Functional Rating Scale-Revised (ALSFRS-R) overall score by week in evaluable patients (*n* = 13). Treatment ended week 24 (assessment performed at week 25) and follow-up ended week 48. Box plot displays median (IQR). Whiskers ( −) are the minimum value; whiskers ( +) are the maximum value. The mean value is indicated by a plus sign. Green dots are slow progressor patients with an ALSFRS-R slope less than − 0.8 points/month. Orange dots are normal progressor patients with an ALSFRS-R slope between − 0.8 and − 1.33 points per month. Red dots are fast progressor patients with an ALSFRS-R slope greater than − 1.33 points/month. IQR, interquartile range

The median (IQR) ALSFRS-R overall score at baseline was 42.0 (37.0, 44.0) and declined progressively throughout the study (Fig. 2). Significant changes from baseline in median ALSFRS-R overall scores were observed at treatment end (week 25, *n* = 11) (− 4.0; *p* = 0.0010) and end of follow-up (week 48, *n* = 10) (− 10.0; *p* < 0.0001) (Table 2). The median (IQR) ALSFRS-R slope was − 0.7 (− 1.2, − 0.5) at treatment end and was − 0.8 (− 1.0, − 0.6) at end of follow-up.

**Table 2:** Change in Amyotrophic Lateral Sclerosis Functional Rating Scale-Revised overall score and subdomain scores and in forced vital capacity, at treatment end (assessment performed at week 25) and end of follow-up in the evaluable population

Parameter	Baseline (*n* = 13)	Week 25 (*n* = 11)		Week 48 (*n* = 10)	
		Change from baseline	*p* value	Change from baseline	*p* value
ALSFRS-R overall, median (IQR)	42.0 (37.0, 44.0)	− 4.0 (− 8.0, − 3.0)	**0.0010**	− 10.0 (− 14.0, − 7.0)	** < 0.0001**
ALSFRS-R bulbar, median (IQR)	10 (9.0, 12.0)	0.0 (− 2.0, 0.0)	0.2500	2.0 (− 4.0, 0.0)	**0.0313**
ALSFRS-R fine and gross motor, median (IQR)	19.0 (17.0, 21.0)	− 3.0 (− 5.0, − 3.0)	**0.0010**	− 7.0 (− 9.0, − 5.0)	** < 0.0001**
ALSFRS-R respiratory, median (IQR)	12.0 (12.0, 12.0)	0.0 (− 2.0, 0.0)	0.1875	0.0 (− 4.0, 0.0)	0.0625
FVC (L), median (IQR)	3.9 (3.0, 4.9)	− 0.4 (− 0.8, 0.0)	**0.0413**	− 0.9 (− 1.8, − 0.3)	**0.0155**
FVC as % of predicted, median (IQR)	87.0 (76.0, 96.0)	− 9.0 (− 23.0, − 6.0)	**0.0068**	− 23.0 (− 38.0, − 9.0)	**0.0006**

ALSFRS-R scores: overall score ranges from 0 (worst) to 48 (best). Lower scores indicate worse ability; higher scores indicate better ability. *p*-values in bold stands for *p* < 0.05

*ALSFRS-R*, Amyotrophic Lateral Sclerosis Functional Rating Scale- Revised; *FVC*, forced vital capacity; *IQR*, interquartile range

Individual subject data for ALSFRS-R overall score indicated a slower than expected decline (less than − 1 point/month) in seven patients (63.4%) at treatment end and in five patients (50.0%) at end of follow-up. Among patients categorized as slow progressors (ALSFRS-R slope less than − 0.8 points/month), the change from baseline to treatment end in median (IQR) ALSFRS-R overall score and median (IQR) ALSFRS-R slope was − 3 (− 3.5, 3) and − 0.5 (− 0.6, − 0.5), respectively. Corresponding values at the end of follow-up were − 7 (− 8, − 6) and − 0.6 (− 0.7, − 0.5).

Based on disease progression between symptom onset and study baseline, five patients (38.5%) were categorized as slow progressors, three (23.0%) as normal progressors and five (38.5%) as fast progressors at study start. Post hoc analysis indicated that six patients (54.5%) maintained their slope progression category (four as slow progressors, one as a normal progressor and one as a fast progressor), and four patients (36.4%) converted to a better slope progression category (two from normal to slow progressor, one from fast to normal progressor and one from fast to slow progressor) at treatment end.

Significant decreases (indicating increased impairment) from baseline to week 48 were observed in median scores for the ALSFRS-R subdomains of bulbar and fine/gross motor function but not respiratory function (Table 2).

Results in the per-protocol population were consistent with those observed in the evaluable population (data not shown).

### Pulmonary function

Median (IQR) FVC and FVC (%) at baseline were 3.9 (3.0, 4.9) L and 87.0% (76.0, 96.0), respectively. Significant decreases from baseline in pulmonary function, as measured by FVC, were observed at treatment end (− 0.4 L; *p* = 0.0413) and end of follow-up (− 0.9 L; *p* = 0.0155) (Table 2). Median changes in FVC (%) of − 9.0% (− 23.0, − 6.0) at treatment end (*p* = 0.0068) and − 23.0% (− 38.0, − 9.0) at end of follow-up (*p* = 0.0006) were statistically significant versus baseline (Table 2). A similar pattern was observed in the per-protocol analysis (data not shown).

### Secondary efficacy endpoints

No significant changes were recorded in behavioural status, current symptom status or cognitive function during the study, as assessed by the ALS-CBS questionnaire (Table 3). No significant changes were seen in ALSAQ-40 scores for communication or emotional functioning, although significant increases (indicating a decrease in quality of life) were observed in the domains of physical mobility, independence and eating/drinking (Table 3).

**Table 3:** Changes in cognitive function/behaviour and quality of life at treatment end (assessment performed at week 25) and end of follow-up in the evaluable population

Parameter	Baseline (*n* = 13)	Week 25 (*n* = 11)		Week 48 (*n* = 10)	
		Change from baseline	*p* value	Change from baseline	*p* value
ALS-CBS					
Behaviour status score, median (IQR)	41.0 (38.0, 42.0)	0.0 (− 3.0, 2.0)	0.7031	− 1.0 (− 6.0, 1.0)	0.2158
Current symptom status score, median (IQR)	3.0 (2.0, 4.0)	0.0 (− 1.0, 1.0)	0.7560	0.0 (− 1.0, 1.0)	0.8281
Cognitive screening score, median (IQR)	17.0 (16.0, 18.0)	0.0 (− 2.0, 1.0)	0.8897	− 1.0 (− 3.0, 2.0)	0.3635
ALSAQ-40					
Physical mobility, median (IQR)	20.0 (12.5, 70.0)	10.0 (0.0, 20.0)	**0.0391**	32.5 (7.5, 50.0)	**0.0015**
Independence, median (IQR)	37.5 (20.0, 67.5)	12.5 (− 2.5, 45.0)	**0.0413**	25.0 (7.5, 45.0)	**0.0048**
Eating/drinking, median (IQR)	0.0 (0.0, 16.7)	0.0 (0.0, 25.0)	0.0625	8.3 (0.0, 58.3)	**0.0313**
Communication, median (IQR)	42.9 (0.0, 57.1)	0.0 (0.0, 28.6)	0.1250	0.0 (0.0, 35.7)	0.0625
Emotional functioning, median (IQR)	25.0 (20.0, 32.5)	5.0 (− 2.5, 22.5)	0.0617	7.5 (0.0, 20.0)	0.0767

*p*-values in bold stands for *p* < 0.05

*ALSAQ-40*, Amyotrophic Lateral Sclerosis Assessment Questionnaire 40; *ALS-CBS*, Amyotrophic Lateral Sclerosis-Cognitive Behavioral Screen; *IQR*, interquartile range

**Fig. 3:**
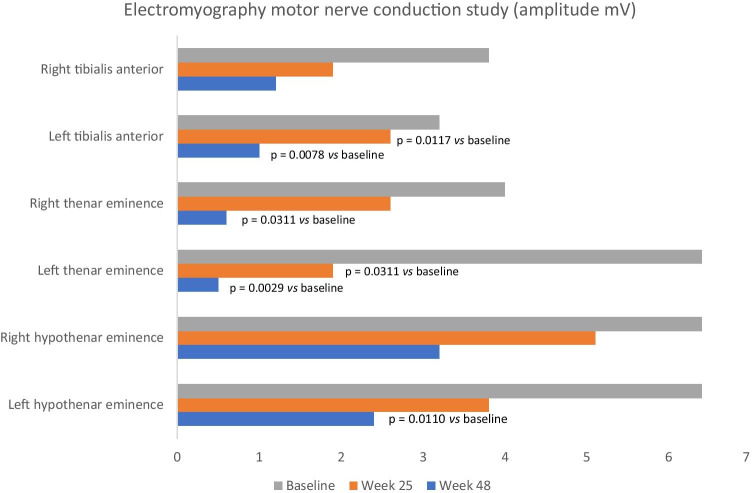
Electromyography motor nerve conduction study results for amplitude (mV) at baseline, treatment end (assessment performed week 25) and end of follow-up in evaluable patients

Compound muscle action potential (CMAP) amplitude decreased from baseline to final visit in the tibialis anterior (TA), thenar eminence (APB) and hypothenar eminence (ADM) on both the right and left sides, indicating axonal loss (Fig. 3). Statistically significant decreases were observed in the left TA, right/left APB and left ADM. No significant changes were observed in distal motor latency (data not shown).

### Biomarkers

**Fig. 4:**
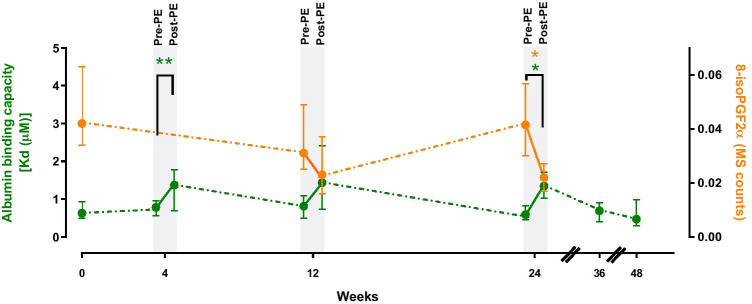
Albumin fatty acid binding capacity and lipid peroxidation by week in evaluable patients (*n* = 13). Treatment ended at week 24 (assessment performed at week 24) and follow-up ended at week 48. 8-isoPGF2α, 8-iso-prostaglandinF2α; Kd, dissociation constant; MS, mass spectrometry; PE, plasma exchange

The total albumin concentration increased significantly during treatment, especially during the initial intensive phase, increasing from 42.70 mg/mL at baseline to 49.45 mg/mL at week 4. After treatment completion and follow-up, albumin concentrations returned to baseline values (42.63 mg/mL). Immediately after PE-A sessions at weeks 4, 12 and 24, albumin showed a lower binding capacity (higher K_d_ value indicating lower affinity) which returned to baseline level during follow-up (weeks 36 and 48) (Fig. 4). A similar profile was observed in 8-isoPGF2α levels, which were decreased immediately after PE-A sessions at weeks 4, 12 and 24 (Fig. 4). A weak but significant correlation was found between the fatty acid binding capacity of albumin and 8-isoPGF2α levels (Spearman’s *R* =  − 0.4; *p* < 0.01).

### Safety

Twelve patients (92.3%) experienced at least one treatment-emergent adverse event (TEAE). Two patients (15.4%) experienced a total of three adverse events (dizziness, presyncope, diarrhoea) potentially related to albumin solution, and two patients (15.4%) experienced a total of three events (headache, presyncope, diarrhoea) potentially related to the PE-A procedure. Of 330 PE procedures, 4.9% were temporally associated with adverse events, and 0.9% were associated with adverse events potentially related to study procedures. Most frequent among 37 reported TEAEs were infections (*n* = 10, none related) and nervous system events (*n* = 6, three related). Most adverse events (86.5%) were mild in intensity. Three patients experienced serious adverse events, none of which was deemed treatment-related. One patient withdrew from the study due to an adverse event (bilateral pneumonia), and one patient died during follow-up from respiratory failure due to disease progression. No clinically significant abnormalities in laboratory parameters were observed.

## Discussion

ALS is a rapidly progressing neurodegenerative disease with limited treatment options beyond symptomatic relief. The most well-established drug with disease-modifying activity is riluzole, which has a modest effect on survival [37, 38]. Although the mechanism of action of riluzole remains unclear, it may include modulation of glutamatergic transmission [39]. Edaravone, which is thought to act as an antioxidant, shows benefit only in a specific subset of patients with early diagnosed ALS and preserved respiratory function [39–41]. As many other molecules have been evaluated but failed to demonstrate clinical efficacy in ALS, a need remains to identify effective treatments [15].

The results of this pilot study suggest that PE-A 5% may be useful in patients with ALS. The study enrolled a relatively young population of patients with a recent diagnosis of ALS and a balanced proportion of bulbar or spinal disease onset. None of the patients had familial ALS or C9orf72 expanded mutation (data not shown). Patients maintained treatment with riluzole throughout the study.

Although functional impairment declined over the 48-week study period, the median change from baseline in the ALSFRS-R overall score (− 4 and − 10 points at weeks 25 and 48, respectively) was less than that expected for natural progression of ALS (− 6 and − 12 points, respectively) [21], and the rate of decline in the ALSFRS-R overall score was slower than expected in seven patients (63.4%) at treatment end and in five patients (50.0%) at end of follow-up. A post hoc comparison of individual patient outcomes according to baseline ALSFRS-R progression slope category showed that 54.5% of patients maintained and 36.4% of patients improved their progressor status at treatment end. These findings are interesting, although likely less relevant for slow progressors.

Evaluation of ALSFRS-R subdomains showed significant declines in bulbar function and fine/gross motor function during the study, whereas respiratory function remained stable. Conversely, the significant decrease observed in FVC suggested that respiratory function declined. The discrepancy between these outcome measures might be explained by the high level of respiratory function in patients at enrolment. Despite the significant decrease in median predicted FVC from 87% at baseline to 71% at final visit, FVC (%) remained above the study inclusion threshold (70%) and above the centre’s threshold (50%) for considering bi-level positive airway pressure interventions. As only three patients (27.3%) at treatment end and five patients (45.5%) at study end had FVC (%) values below 50%, respiratory dysfunction was not pronounced in the study population. The discordance in outcome measures may also reflect the proportion of patients (53.8%) with bulbar disease at baseline as these patients frequently have difficulty performing lung function tests correctly.

The results of the ALSAQ-40 questionnaire and EMG recordings supported the observation that patients’ condition worsened over time. In contrast, no changes in ALS-CBS scores were observed over the course of the study. Frontotemporal dementia is found in approximately 14% of patients with ALS, and more than 40% of ALS patients have evidence of cognitive impairment without dementia [42]. The baseline median ALS-CBS score of 17 in our cohort is consistent with no clear cognitive impairment [43]. Previously, it has been shown that cognitive deterioration is less likely in ALS patients who have normal cognition at baseline [44].

Biomarker analyses suggested that PE-A produced transient changes which returned to baseline values at treatment end. The changes observed in fatty acid albumin binding capacity may reflect binding by albumin to lipidic substances mobilized during PE. The lower 8-isoPGF2α levels observed after each session is consistent with higher occupation of albumin fatty acid binding sites since fatty acid binding site 1 has been described as the main binding site for prostaglandins [45]. However, whether the decrease observed in lipid peroxidation was due to reduced albumin binding or was consequential to direct removal by PE remains unclear. In our understanding, this observation and the role of albumin in ALS pathology warrant further investigation.

Consistent with the results of previous studies [18–20], PE-A was safe and feasible in this cohort of ALS patients. Only 0.9% of exchange procedures were associated with adverse events judged by investigators as being potentially related to albumin and/or the PE procedure and all were mild in severity. A shortcoming of PE is that, at minimum, sustainable venous access is required to perform periodic procedures during the course of a study which might be considered invasive and is burdensome for the patient. In our study, peripheral venous access was used for all patients, and as such, the procedure was considered tolerable.

The main limitations of this pilot study are the modest sample size and phenotypic variability in ALS which was not controlled. The results must therefore be considered as preliminary. As the study was not powered for efficacy, we could conclude only that a trend was evident for PE-A to be safe and feasible in our ALS cohort. Given the heterogeneity of progression in ALS [46], we used the change in individual ALSFRS-R total scores as a linear measure of progression. Although a subject of debate, this approach been used successfully in a recent study [47]. The observation that some patients showed a slower than expected decline in their ALSFRS-R overall score and that some patients improved their progressor status at treatment end is intriguing. Whether these outcomes are attributable to a relatively young patient population, heterogeneity in normal disease evolution or reflect a beneficial effect of PE-A requires further research. A twin pilot study currently underway in the USA may offer further insight (ClinicalTrials.gov identifier: NCT02872142) [48].

In conclusion, this pilot study suggests that PE with albumin 5% (Albutein® 5%) replacement has an acceptable safety profile in patients with ALS. The observation that 63.4% of patients showed a slower than expected decline in their ALSFRS-R score at treatment end and that 36.4% of patients showed a reduction in ALSFRS-R slope progression during treatment provides a rationale for additional research in this underserved therapeutic setting.

## Data Availability

Data reported in this manuscript are available within the article and/or its supplementary materials and/or clinical trials databases (EudraCT#: 2013–004,842-40; ClinicalTrials.gov ID: NCT02479802). Additional data are available from the corresponding author upon reasonable request.
